# A Human Monoclonal Antibody against Hepatitis B Surface Antigen with Potent Neutralizing Activity

**DOI:** 10.1371/journal.pone.0125704

**Published:** 2015-04-29

**Authors:** Antonella Cerino, Corinna M. Bremer, Dieter Glebe, Mario U. Mondelli

**Affiliations:** 1 Research Laboratories, Department of Infectious Diseases, Fondazione IRCCS Policlinico San Matteo, Pavia, Italy; 2 Institute of Medical Virology, National Reference Centre for Hepatitis B and D Viruses, German Center for Infection Research, Justus-Liebig University of Giessen, Giessen, Germany; 3 Department of Internal Medicine and Therapeutics, University of Pavia, Pavia, Italy; CRCL-INSERM, FRANCE

## Abstract

We describe the production and characterization of human monoclonal antibodies (mAb) specific for the major hepatitis B virus (HBV) S protein. The mAbs, two IgG1κ and one IgG1λ, were secreted by B-cell clones obtained from peripheral blood mononuclear cells (PBMC) of one person convalescent from acute hepatitis B and one vaccinated individual. The former recognized a denaturation-insensitive epitope within the p24 protein whereas the latter recognized a denaturation-sensitive, conformational epitope located within the HBsAg common “a” determinant. This mAb, denominated ADRI-2F3, displayed a very high protective titer of over 43,000 IU/mg mAb and showed an extremely potent neutralizing activity in the in vitro model of HBV infection using primary hepatocytes from *Tupaia belangeri* as target. Recombinant variable heavy and light domain sequences derived from mAb ADRI-2F3 were cloned into eukaryotic expression vectors and showed identical fine specificity and 1 log_10_ higher titer than the original IgG1λ. It is envisaged that such mAb will be able to efficiently prevent HBV reinfection after liver transplantation for end-stage chronic HBV infection or infection after needle-stick exposure, providing an unlimited source of valuable protective anti-HBs antibody.

## Introduction

Hepatitis B virus (HBV) is one of the world's most common infectious agents causing millions of infections each year [1]. Between 500,000 and 700,000 people die each year from chronic infection-related cirrhosis, hepatocellular carcinoma (HCC) or from fulminant hepatitis B [1,2]. Transmission occurs via percutaneous and mucosal exposure to infectious body fluids. Therefore, the most common route of transmission is sexual transmission. However, infection through blood transfusions and blood products has not been completely eliminated [3] and contaminated injections during medical procedures, sharing of needles, syringes and paraphernalia among intravenous drug users still represent a major public health problem. Vertical transmission is common, especially in Asia, where HBV titers in maternal blood are high, and in developing countries which have not yet implemented hepatitis B vaccination. Further, HBV poses a risk to healthcare workers exposed to accidental needle-stick injuries.

The currently marketed hepatitis B vaccines contain the major viral envelope protein hepatitis B surface antigen (HBsAg). Vaccination with HBsAg provides protection against HBV infection and prevents complications including liver cirrhosis and HCC [4]. The control and the eventual elimination of HBV infection are possible with the appropriate use of hepatitis B vaccines, and this will reduce significantly the disease burden and its associated costs.

Although prevention of HBV infection may be effectively achieved by vaccination there are certain situations that require a different prophylactic approach. Liver transplantation for end-stage HBV-related liver disease is one such example. Hepatitis B immune globulin (HBIG) has played a central role in prophylaxis against recurrent hepatitis B in patients undergoing liver transplantation.

Prior to the routine use of HBIG as immunoprophylaxis, recurrence of HBV in the liver allograft occurred in up to 80%, and infrequently was associated with an aggressive fibrosing cholestatic variant that caused progressive graft dysfunction and significant mortality. The subsequent availability of safe and effective antiviral drugs led to additional survival benefits by improving prophylactic efficacy and preventing disease progression in those with recurrence [5].

HBIG is a polyclonal antibody to HBV surface antigen (HBsAg) derived from pooled human plasma. Although its mechanism of action is not yet completely understood, it is thought that HBIG acts in the circulation by preventing hepatocyte infection, binding to and neutralizing circulating virions expressing HBsAg and perhaps inducing lysis of infected cells [6]. Within the liver, HBIG may also prevent cell-to-cell infection as well as reduce HBsAg and virion secretion upon endocytosis into hepatocytes [7].

To provide maximal protection against re-infection of the liver graft, HBIG should be given frequently (typically daily) for the week following transplantation. The pivotal multicenter European trial demonstrated that long term administration of intravenous (IV) HBIG reduced hepatitis B recurrence rates from 75% to 36% and was associated with improved graft and patient survival [8]. Subsequent trials, using variable schedules for HBIG administration, confirmed the efficacy of HBIG as a monotherapy against recurrent HBV infection [9].

HBIG prophylaxis is expensive. HBIG is commonly administered intravenously at high dose, daily for the first week and monthly thereafter, which makes the current costs of management of patients transplanted for HBV-related cirrhosis prohibitive, even for developed countries. Dose reduction has been proposed for cost reduction, either based on a flat dose or on a response-guided basis in order to maintain circulating anti-HBs at a protective level. However, HBIG doses are variable and should be individualized among patients. It has also been proposed to abandon HBIG prophylaxis in favor of using antiviral drugs alone, however this is a very controversial issue [5].

The costs of HBIG treatment and prevention are not the only limitations to its use. Additional limitations include the following: i) supply is limited and depend on vaccinated human donors exhibiting high titer protective anti-HBs, ii) purification is time consuming and must undergo lengthy virus-inactivation procedures; iii) anti-HBs titer is variable and effective virus neutralization efficiency largely unknown being exclusively based on arbitrarily protective anti-HBs serum titers; iv) polyclonal immunoglobulin include several antibody specificities and may select for HBV mutants resistant to currently available antiviral drugs; v) HBIG preparations are currently combined with antiviral drugs to insure complete protection, thus adding to the costs.

In this paper we describe the production and characterization of human monoclonal antibodies (humAb) specific for the HBV major (S) envelope protein. One of these humAb recognized a conformational epitope located in the common “a” determinant and neutralized HBV with very high efficiency *in vitro* using HBV-susceptible primary hepatocytes from *Tupaia belangeri* (PTH). We believe that such humAb offers many advantages over the commercially available plasma-derived HBIG and should be tested in vivo for protection against HBV reinfection following liver transplantation and as a general post-exposure prophylaxis against hepatitis B.

## Materials and Methods

### Statement

This study was approved by the Institutional Review Board of Policlinico San Matteo, Italy. Participants were administered a written informed consent which they signed, and is conserved in our files. The study complied with the recommendations of the 1975 Declaration of Helsinki.

### Isolation of HBsAg specific B-cell clones

Peripheral blood mononuclear cells (PBMC) were obtained from a vaccinated healthy donor (ADRI) and from a person convalescing from acute hepatitis B (PK). Both subjects showed high serum anti-HBs titers. PBMC were isolated by standard density gradient centrifugation and B cells negatively purified using a B cell isolation kit II (code n°130-042-201 MACS- Miltenyi Biotec). B cells (10^6^/ml, purity >95%) were then incubated v/v with undiluted supernatant from the EBV-productive B95-8 marmoset cell line for 24 h at 37°C, 5% CO; washed and incubated in complete media consisting of RPMI-1640 Dutch mod.(code n° R7638 Sigma) supplemented with 10% fetal bovine serum (code n° SH30071.03 HyClone), 4mM L-glutamine (code n°G7513 Sigma), 2mM Na-Pyruvate (code n° S8636 Sigma), 1% MEM non-essential aminoacids 100X (code n° M7145 Sigma), 1% antibiotic-antimycotic solution (code n° A5955 Sigma), 2.5 μg/ml CpG-2006 (Microsynt) and 10 ng/ml IL-2 (code n° 202-IL R&D Systems) as described [10], with minor modifications which consisted in omitting enrichment of CD27^+^ memory B cells. 3 x 10^3^ B cells in complete media were then seeded in each well of a 96-well round-bottom plate (code n° 3799 Costar) in the presence of irradiated feeder cells and incubated for 5 days after which IL-6 was added at a final concentration of 20 ng/ml. After evident visual outgrowth of lymphoblastoid cells, supernatants were tested by ELISA for the presence of anti-HBV envelope antibodies using a recombinant HBsAg preparation. Cultures exhibiting a 492 nm optical density >1 were cloned and subcloned by limiting dilution in complete media + 20 ng/ml IL-6 in the presence of irradiated feeder cells to obtain stable Ag-specific B-cell clones. The general procedure to obtain human HCV-specific B-cell clones from PBMC has been extensively described previously [11–14].

### B-cell clone specificity

This was performed by testing B-cell clone supernatants by standard ELISA using a recombinant HBsAg preparation containing pre-S1, pre-S2 and S proteins. Stable clone supernatants were also examined by Western blot using recombinant denatured HBsAg as Ag. Binding to the common “a” determinant on the S polypeptide was determined by a competition ELISA using a murine mAb Hyb-824 (Cosmo Bio Co., LTD. Code n° 2ZHB11 Cosmo Bio Co. LTD.) specific for the same region. Briefly, microtiter plates were coated with HBsAg at a concentration of 1 μg of 0.05M bicarbonate buffer (pH 9.6) per ml at 4°C overnight. After saturation with 0.1M PBS containing 2% bovine serum albumin (BSA). Murine monoclonal antibody Hyb-824 was diluted to a concentration of 200 ng/well and incubated at increasing concentrations (0 ng/w—400 ng/w) of human monoclonal antibodies ADRI-2F3, and its corresponding recombinant recADR12F3, PK3D1 and PK10C7 for 1 h at 37°C. Plates were washed with PBS + 0.05% Tween20, then 100 μl of diluted HRP- AffiniPure- goat anti-mouse IgG (H+L) (code 115-035-062 Jackson-ImmunoResearch) in 2% BSA-PBS were added and incubated for 1 h at 37°C. After washing again with PBS + 0.05% Tween20, 100 μl of OPD solution (code S2045 DAKO) were added and the O.D. measured at 492nm. Humab anti-HCV CM3-B6 and anti-HBV preS1 MA18/7 were used as negative control, whereas Hyb824 alone was used as positive control.

### HBV neutralization assay

This was performed as extensively described [15]. Briefly, primary *Tupaia belangeri* hepatocyte cultures (PTH) which had been shown to be susceptible to HBV both *in vivo* and *in vitro* [16] were inoculated with purified HBV from human serum at a ratio of 100 HBV genome/hepatocyte. Permission to use primary hepatocytes from *Tupaia belangeri* was obtained from the Institutional Review Board of Justus-Liebig University of Giessen and animals were handled according to ethical rules for animals care. To determine whether infection of PTH by HBV could be prevented by human anti-HBs mAbs, HBV was preincubated with three different human mAbs ADRI-2F3, PK10C7, PK3D1, at dilutions ranging from 1:10^1^ to 1:10^6^. The neutralization power of the human anti-HBs mAbs was directly compared with a well-characterized preS1-specific antibody (MA18/7) which was previously shown to exhibit significant neutralizing activity in PTH *in vitro* infection assay [15]. The read-out of successful HBV-infection of these cultures was newly produced HBsAg 11 to 15 days after infection. Neutralization was complete, if no viral protein production was detectable in the culture supernatant [15].

### 5′ Rapid Amplification of cDNA Ends (RACE) and sequencing of mAb ADRI-2F3 variable domains

Cloning and sequencing were performed by ModiQuest Research (Oss, The Netherlands) using proprietary material and procedures. Briely, total RNA extracted from pelleted viable B-cell clone cultures treated with Trizol-containing buffer. The coding sequences for the variable heavy and light domains were determined by means of 5′ RACE using proprietary vectors developed by ModiQuest Research.

### Cloning and eukaryotic production of recombinant ADRI-2F3 hIgG1λ

Variable heavy and light domain sequences obtained as described above, were cloned into eukaryotic expression vectors pMQR-hIgG1 and pMQR-hIgλ (ModiQuest Research, proprietary procedure). Recombinant antibodies were produced in transiently transfected Hek293 cells (ModiQuest Research, proprietary procedure). Next, recombinant spent culture supernatant from recombinant ADRI-2F3 mAb was validated against HBsAg in ELISA in duplicate using supernatant from the original B-cell clone as comparator. To this end, HBsAg was coated in carbonate buffer in an ELISA plate O/N at 4 C°. Following blocking, serial dilutions were allowed to bind at room temperature in duplicate: recombinant ADRI-2F3-13132 culture sup., human mAb anti-HBs ADRI-2F3, and complete medium as negative control. Plate was washed and bound Ab visualized using goat anti-human IgG-HRP and TMB (extinction was read at 450 nm).

## Results

### Ag-specificity of human mAbs

**Fig 1:**
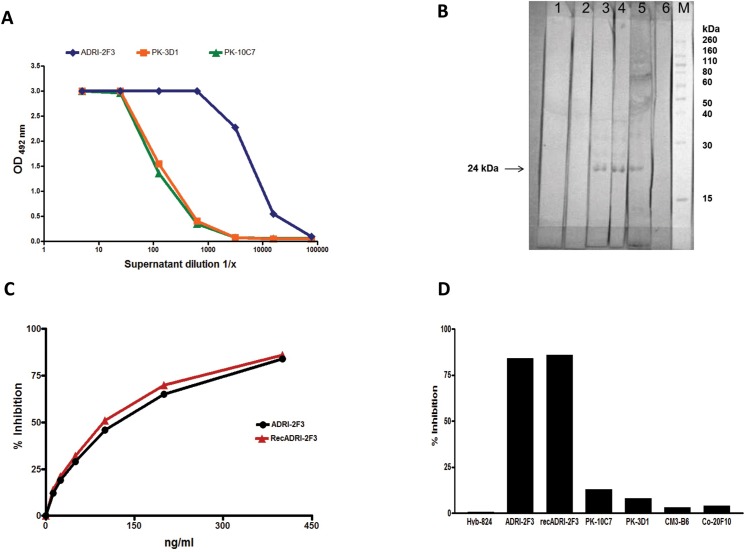
A) Titration curve of humAb binding to HBsAg in an ELISA. Diamonds: ADRI-2F3, Squares: PK-3D1, Triangles PK-10C7. B) Western blot showing reactivity of humAb ADRI-2F3 (lanes 1 and 2), PK-10C7 (lane 3) and PK-3D1 (lane 4). Lane 5; positive control serum, lane 6; negative control. MW indicates a molecular weight marker. Note that in lanes 3, 4 and 5 a 24 kDa band is recognized, indicating binding to the HBsAg polypeptide. C) Competitive inhibition of serial dilutions (0 ng/ml—400 ng/ml) of humAb ADRI-2F3, and its corresponding recombinant recADR12F3, binding to HBsAg by 200 ng of murine mAb Hyb-824 specific for the HBsAg common “a” determinant. D) Competitive inhibition of humAb ADRI-2F3, recADRI2F3, PK-10C7, PK-3D1 by murine mAb Hyb-824. CM3B6, a HCV-NS3 specific humAb, served as negative control. Co20-F10 is an isotype control.

Three human B-cell clones (ADRI-2F3, PK10C7, PK3D1) and several subclones were derived from the two subjects mentioned in Materials and Methods. All humAb IgG showed strong binding to HBsAg in ELISA (Fig 1A). ADRI-2F3 secreted IgG1λ, whereas PK10C7 and PK3D1 secreted IgG1κ. Western blot analysis showed that PK10C7 and PK3D1 recognized a sequential, denaturation-insensitive epitope located within a band corresponding to the expected molecular weight (24 kDa) of the major S envelope protein [17], whereas ADRI-2F3 did not bind to any protein band, indicating that it recognized a denaturation-sensitive conformational epitope (Fig 1B). Competitive ELISA showed that ADRI-2F3 and its recombinant recADRI2F3 binding to HBsAg was almost completely inhibited by addition of murine mAb specific for the HBsAg common “a” determinant (Fig 1C & 1D) while PK10C7 and PK3D1 binding to HBsAg was not significantly affected by the same mAb (Fig 1D)

### mAb neutralizing activity

**Fig 2:**
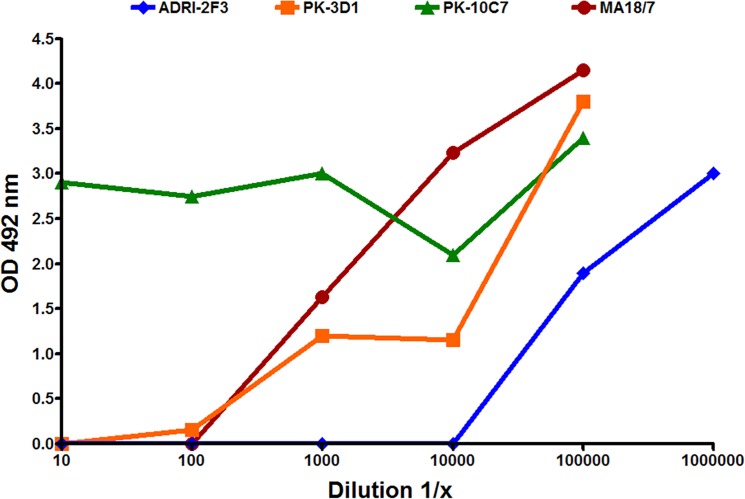
Neutralizing activity of three human monoclonal antibodies PK-3D1, PK-10C7 and ADRI-2F3 in comparison with mouse monoclonal antibody MA18/7. It is shown herein that a dilution of 1:10,000 of monoclonal antibody supernatant ADRI-2F3 completely neutralizes the activity of HBV, whereas EC50 is reached at a dilution of 1: 100,000.

This was analyzed for both ADRI- and PK-derived humAb using the neutralization assay described above. ADRI-2F3 supernatant showed strong neutralization activity up to 1:10,000 dilution. Since the specific IgG1λ concentration in the supernatant was 27 μg/ml it can be extrapolated that ADRI-2F3 efficiently neutralized HBV at concentrations of at least 2.7 ng/ml (Fig 2). Since the viral inoculum used for infection of PTH was 10^8^/ml it can be derived that no more than 2.7 ng/ml were sufficient to neutralize 10^8^ HBV particles. PK humAb supernatant showed contrasting behaviors. Thus, PK10C7 did not neutralize infection of PTH while PK3D1 showed moderate neutralizing activity down to a dilution of 1:10^2^ (about 200 ng/ml) which proved to be at least as good as the standard murine MA18/7 anti-pre-S1 mAb used as positive neutralization standard (1:100, corresponding to a concentration of 10 μg/ml) (Fig 2).

### Sequencing of mAb ADRI-2F3 variable domains

Since ADRI-2F3 showed potent HBV neutralizing activity, 38 cDNA VH sequences and 36 cDNA VL sequences were obtained from 19 clones (IGHV3‐23*01, IGHD6‐19*01, IGHJ2*01) and 18 clones (IGLV3‐21*02, IGLJ7*01), respectively, from ADRI-2F3 only. The resulting VH and VL domains were sequenced (in sense and in antisense direction) of n = 20 VH and n = 20 VL cDNA clones of the 5′ RACE reaction on RNA isolated from human B‐cell clone ADRI-2F3 which yielded clear sequences for both the VH and the VL domains (n = 38 and 36 identical sequences, respectively). Few individual point mutations were detected in the sequence files of individual clones. These ambiguities were solitary occurrences in an otherwise unambiguous sequence alignment, most likely caused by erroneous insertion of bases during the polymerase chain reaction, or a bias in sequencing reactions. Sequencing was performed in opposite directions; i.e. two sequencing reactions were obtained per clone. Only full‐length cDNAs with an obvious sequence were analyzed. Germline alignment allowed identification of complementarity determining regions (CDRs) which determine the specificity for HBsAg. VH and VL sequences from clone ADRI-2F3 are available as S1 Fig.

### Eukaryotic expression of ADRI-2F3 hIgG1λ yielded a recombinant with specificity identical to the original clone

**Fig 3:**
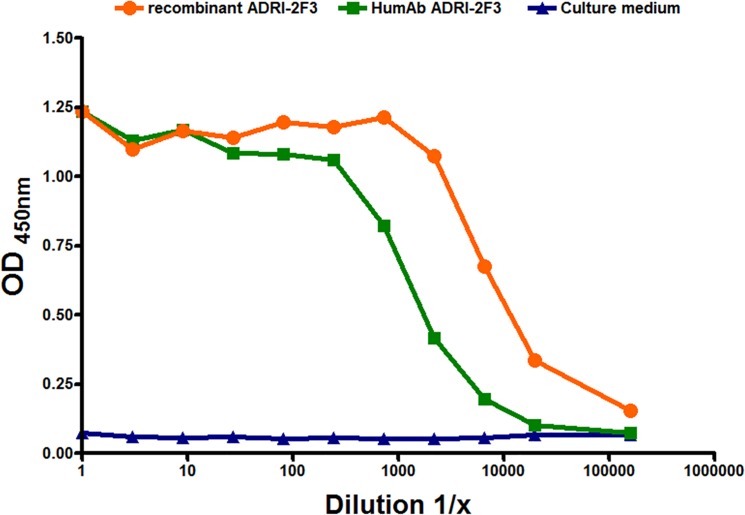
Reactivity of recombinant antibody ADRI-2F3 versus human monoclonal ADRI-2F3. The titration curve shows that the recombinant antibody is about 10 times more reactive than the original human monoclonal antibody.

As VH and VL sequences were all identical, representative VH and VL sequences were cloned in the eukaryotic expression vector described above. Recombinant ADRI-2F3 proved to be reactive towards its target antigen. Interestingly, the recombinant ADRI-2F3 spent culture supernatant displayed a reactivity of approximately one log_10_ scale above the spent culture supernatant of the originating human B‐cell clone, as detailed in Fig 3. Therefore this experiment conclusively showed that recombinant ADRI-2F3 could be cloned, produced, and reactivity towards its target antigen (HBsAg) could be validated by ELISA.

## Discussion

There is an unmet medical need for sustainable reagents allowing standardization of immunoprophylaxis of HBV re-infection in the liver transplant setting. Such a standardized reagent could also beneficially be used in other settings, such as in the acute treatment of accidental needle-sticks and in the prevention of vertical-perinatal HBV transmission. Administration of anti-HBs antibodies is usually performed as a prophylactic measure while waiting for the full development of vaccine-induced adaptive immunity to HBV. To this end, several mAb have been proposed and developed in order to address that need. Most mAb against HBV are of murine origin. Such murine antibodies have the inherent disadvantage that they evoke an immune reaction in a human recipient when used in a therapeutic or prophylactic composition. HumAb capable of neutralizing HBV infection have also been described. For instance two humAb termed HBV-17 and HBV-19, obtained from PBMC from HBV vaccinated individuals engrafted in the Trimera mouse model were described with a specific activity of 514 IU/mg and 2,900 IU/mg respectively [18]. Another series of humAb has been described by Tajiri et al., [19]. These humAb were derived from human VH/VL chain fragments from B cells of vaccinated persons and cloned in a vector. However, they did not seem to provide full protection against HBV infection *in vitro*, even not at the highest concentrations tested providing still residual activity after neutralization of HBV with each of the humAb tested. Another humAb HB-C7A has been described by Shin et al. which was able of fully preventing HBV infection in two chimpanzees after mixing 100 CID50 of HBV and 100 μg of HB-C7A [20–21]. The humAb was constructed from a phage-displayed Ab library and converted into a human IgG1. It has been reported to have a titer of 2,600 IU/mg.

Here we report the generation of three fully human mAb obtained by cloning B cells from one patient convalescent from acute hepatitis B and one vaccinated individual which recognize the major S protein of HBV. The latter shows an extremely high protective anti-HBs titer and recognizes a conformational epitope within the common “a” determinant of HBsAg. Most importantly, it shows a very high neutralization capacity in an *in vitro* HBV infection system using *PTH* as target. The potency of the humAb (43,000 IU/mg of Ig) exceeds by >1 log_10_ that of the best of the previously described mAb HB-C7A (2,600 IU/mg of Ig). Moreover, ADRI-2F3 is a fully human IgG1λ directly produced and secreted by a B-cell clone without prior variable chain fragment cloning or construction of a phage-displayed Ab library.

One potential problem in the prevention of HBV infection or reinfection is the emergence of HBsAg escape mutants, which have been described in a minority of patients on HBIG prophylaxis after liver transplantation or emerging as a result of selection after immunization [22, 23]. This problem can by and large be overcome by maintaining a high titer of neutralizing antibody and this can be achieved with a potent mAb such as the one described herein. The problem may be circumvented by preparing pools of mAbs, although this would be extremely difficult to achieve working with human mAbs since the common “a” determinant is a dominant epitope which tends to monopolize the specificity of immune responses to a complex protein. Indeed, the “original antigenic sin”, first described in 1953 [24], is the phenomenon in which sequential exposure to viral variants induces preferential antibody response to a dominant virus strain encountered in the past. As a result, the response to the minor strains is diminished.

The humAb presented herein and in particular ADRI-2F3 may advantageously be used to treat or prevent HBV infection, as it neutralizes HBV very efficiently and effectively. Due to its extreme potency, the mAb may be administered at very low quantities, thus contributing to a cost-effective treatment. In general, the antibody may preferably be administered to humans in order to maintain a serum level of between 100 and 200 IU/L serum which is considered protective to avoid reinfection of the engrafted liver. The conventional protective titer of anti-HBs in vaccinated persons is arbitrarily set at 12 IU/L. Moreover, ADRI-2F3 variable domains may advantageously be used to produce an unlimited amount of humAb, without relying on vaccinated donor plasma, as well as recombinant antibodies or other binding molecules effective in the treatment or prevention of HBV infection. It would be desirable that such mAb produced under GMP conditions be used in clinical trials of prophylaxis of HBV reinfection following liver transplantation or prevention of HBV infection following needle-stick exposure.

## Supporting Information

S1 Fig ADRI-2F3 variable heavy (VH) and light (VL) chain sequences. (ODT)
