# Supplementary material for: A Human Monoclonal Antibody against Hepatitis B Surface Antigen with Potent Neutralizing Activity
Source: PLoS One. 2015 Apr 29;10(4):e0125704. doi: 10.1371/journal.pone.0125704 (PMC4414269; doi:10.1371/journal.pone.0125704)
Supplement: Supplementary file 1 [file pone.0125704.s001.odt]

ADRI-2F3-VH

(130814GJ)

	  W  L  F   L  V  A  I   L  K  G   V  Q  C   E  V  Q  V   L  E  S   G  G  G   L  V  Q  P   G  G  S   L  R  L

       1	CTGGCTTTTT CTTGTGGCTA TTTTAAAAGG TGTCCAGTGT GAGGTGCAGG TGTTGGAGTC TGGGGGAGGC TTGGTACAGC CGGGGGGGTC CCTGAGACTC

	GACCGAAAAA GAACACCGAT AAAATTTTCC ACAGGTCACA CTCCACGTCC ACAACCTCAG ACCCCCTCCG AACCATGTCG GCCCCCCCAG GGACTCTGAG

	 S  C  A  A   S  G  F   R  F  S   S  Y  A  M   S  W  V   R  Q  A   P  G  K  G   L  E  W   V  S  G   I  S  G  T ·

     101	TCCTGTGCAG CCTCTGGATT CAGGTTTAGC AGCTATGCCA TGAGTTGGGT CCGCCAGGCT CCAGGGAAGG GGCTGGAGTG GGTCTCAGGT ATTAGTGGTA

	AGGACACGTC GGAGACCTAA GTCCAAATCG TCGATACGGT ACTCAACCCA GGCGGTCCGA GGTCCCTTCC CCGACCTCAC CCAGAGTCCA TAATCACCAT

	·  G  E  N   T  Y  Y   A  D  S  V   K  G  R   F  T  I   S  R  D  N   S  K  N   T  L  Y   V  Q  M  N   S  L  R ·

     201	CTGGTGAAAA CACATACTAC GCAGACTCCG TGAAGGGCCG GTTCACCATC TCCAGAGACA ATTCCAAGAA CACGCTGTAC GTGCAAATGA ACAGCCTGAG

	GACCACTTTT GTGTATGATG CGTCTGAGGC ACTTCCCGGC CAAGTGGTAG AGGTCTCTGT TAAGGTTCTT GTGCGACATG CACGTTTACT TGTCGGACTC

	· A  E  D   T  A  V  Y   Y  C  A   K  D  A   I  L  G  S   G  H  P   W  Y  F   H  V  W  G   R  G  T   L  V  T

     301	AGCCGAAGAC ACGGCCGTAT ATTACTGTGC GAAAGATGCG ATCTTGGGCA GTGGCCACCC CTGGTACTTC CATGTCTGGG GCCGTGGCAC CCTGGTCACT

	TCGGCTTCTG TGCCGGCATA TAATGACACG CTTTCTACGC TAGAACCCGT CACCGGTGGG GACCATGAAG GTACAGACCC CGGCACCGTG GGACCAGTGA

	 V  S  S  A   S  T  K   G  P  S   V  F  P  L   A  P  S   S  K  S   T  S  G  G   T  A  A   L  G  C   L  V  K  D ·

     401	GTCTCCTCAG CCTCCACCAA GGGCCCATCG GTCTTCCCCC TGGCACCCTC CTCCAAGAGC ACCTCTGGGG GCACAGCGGC CCTGGGCTGC CTGGTCAAGG

	CAGAGGAGTC GGAGGTGGTT CCCGGGTAGC CAGAAGGGGG ACCGTGGGAG GAGGTTCTCG TGGAGACCCC CGTGTCGCCG GGACCCGACG GACCAGTTCC

DNA sequence:

gaggtgcaggtgttggagtctgggggaggcttggtacagccgggggggtccctgagactctcctgtgcagcctctggattcaggtttagcagctatgccatgagttgggtccgccaggctccagggaaggggctggagtgggtctcaggtattagtggtactggtgaaaacacatactacgcagactccgtgaagggccggttcaccatctccagagacaattccaagaacacgctgtacgtgcaaatgaacagcctgagagccgaagacacggccgtatattactgtgcgaaagatgcgatcttgggcagtggccacccctggtacttccatgtctggggccgtggcaccctggtcactgtctcctca

AA sequence:

EVQVLESGGGLVQPGGSLRLSCAASGFRFSSYAMSWVRQAPGKGLEWVSGISGTGENTYYADSVKGRFTISRDNSKNTLYVQMNSLRAEDTAVYYCAKDAILGSGHPWYFHVWGRGTLVTVSS

ADRI-2F3-VL

(130814GJ)

	                                       NcoI

	                                      ~~~~~~~

	  W  A  *   G  S  S  T   G  G  A   S  A  M   A  W  T  V   L  L  L   G  L  L   S  H  C  T   G  S  V   T  S  Y

       1	CTGGGCCTAA GGAAGCAGCA CTGGTGGTGC CTCAGCCATG GCCTGGACCG TTCTCCTCCT CGGCCTCCTC TCTCACTGCA CAGGCTCTGT GACCTCCTAT

	GACCCGGATT CCTTCGTCGT GACCACCACG GAGTCGGTAC CGGACCTGGC AAGAGGAGGA GCCGGAGGAG AGAGTGACGT GTCCGAGACA CTGGAGGATA

	                                                                                               ApaLI

	                                                                                               ~~~~~~~

	 V  L  T  Q   P  P  S   V  S  V   A  P  G  Q   T  A  R   M  T  C   G  G  N  N   I  G  S   E  S  V   H  W  F  Q ·

     101	GTGCTGACTC AGCCTCCCTC GGTGTCAGTG GCCCCAGGAC AGACGGCCAG GATGACCTGT GGGGGAAACA ACATTGGAAG TGAAAGTGTG CACTGGTTCC

	CACGACTGAG TCGGAGGGAG CCACAGTCAC CGGGGTCCTG TCTGCCGGTC CTACTGGACA CCCCCTTTGT TGTAACCTTC ACTTTCACAC GTGACCAAGG

	                                                                  BamHI

	                                                                  ~~~~~~

	·  Q  K  P   G  Q  A   P  V  L  V   V  Y  D   D  S  D   R  P  S  G   I  P  E   R  F  S   G  S  N  S   G  N  T ·

     201	AGCAGAAGCC AGGCCAGGCC CCTGTGCTGG TCGTCTATGA TGATAGCGAC CGGCCCTCAG GGATCCCTGA GCGATTCTCT GGCTCCAACT CTGGGAACAC

	TCGTCTTCGG TCCGGTCCGG GGACACGACC AGCAGATACT ACTATCGCTG GCCGGGAGTC CCTAGGGACT CGCTAAGAGA CCGAGGTTGA GACCCTTGTG

	· A  T  L   T  I  S  R   V  E  A   G  D  E   A  D  Y  Y   C  Q  V   W  D  S   S  S  D  H   A  V  F   G  G  G

     301	GGCCACCCTG ACCATCAGTA GGGTCGAAGC CGGGGATGAG GCCGACTATT ACTGTCAGGT GTGGGATAGT AGTAGTGATC ATGCTGTGTT CGGAGGAGGC

	CCGGTGGGAC TGGTAGTCAT CCCAGCTTCG GCCCCTACTC CGGCTGATAA TGACAGTCCA CACCCTATCA TCATCACTAG TACGACACAA GCCTCCTCCG

	 T  Q  L  T   V  L  G   Q  P  K   A  A  P  S   V  T  L   F  P  P   S  S  E  E   L  Q  A   N  K  A   T  L  V  C ·

     401	ACCCAGCTGA CCGTCCTCGG TCAGCCCAAG GCTGCCCCCT CGGTCACTCT GTTCCCACCC TCCTCTGAGG AGCTTCAAGC CAACAAGGCC ACACTGGTGT

	TGGGTCGACT GGCAGGAGCC AGTCGGGTTC CGACGGGGGA GCCAGTGAGA CAAGGGTGGG AGGAGACTCC TCGAAGTTCG GTTGTTCCGG TGTGACCACA

DNA sequence:

tcctatgtgctgactcagcctccctcggtgtcagtggccccaggacagacggccaggatgacctgtgggggaaacaacattggaagtgaaagtgtgcactggttccagcagaagccaggccaggcccctgtgctggtcgtctatgatgatagcgaccggccctcagggatccctgagcgattctctggctccaactctgggaacacggccaccctgaccatcagtagggtcgaagccggggatgaggccgactattactgtcaggtgtgggatagtagtagtgatcatgctgtgttcggaggaggcacccagctgaccgtcctc

AA sequence:

SYVLTQPPSVSVAPGQTARMTCGGNNIGSESVHWFQQKPGQAPVLVVYDDSDRPSGIPERFSGSNSGNTATLTISRVEAGDEADYYCQVWDSSSDHAVFGGGTQLTVL
